# Enhanced Recovery after Bariatric Surgery: Systematic Review and Meta-Analysis

**DOI:** 10.1007/s11695-016-2438-z

**Published:** 2016-11-06

**Authors:** Piotr Małczak, Magdalena Pisarska, Major Piotr, Michał Wysocki, Andrzej Budzyński, Michał Pędziwiatr

**Affiliations:** 1 2nd Department of General Surgery, Jagiellonian University Medical College, Krakow, Poland; 2 Department of Endoscopic, Metabolic and Soft Tissue Tumors Surgery, Kopernika 21, 31-501 Kraków, Poland

**Keywords:** ERAS, Bariatric surgery, Gastric bypass, Sleeve gastrectomy

## Abstract

Enhanced recovery after surgery (ERAS) protocol is well established in many surgical disciplines and leads to a decrease in the length of hospital stay and morbidity. Multimodal protocols have also been introduced to bariatric surgery. This review aims to evaluate the current literature on ERAS in obesity surgery and to conduct a meta-analysis of primary and secondary outcomes. MEDLINE, Embase, Scopus and Cochrane Library were searched for eligible studies. Key journals were hand-searched. We analysed data up to May 2016. Eligible studies had to contain four described ERAS protocol elements. The primary outcome was the length of hospital stay; the secondary outcomes included overall morbidity, specific complications, mortality, readmissions and costs. Random effect meta-analyses were undertaken. The initial search yielded 1151 articles. Thorough evaluation resulted in 11 papers, which were analysed. The meta-analysis of the length of stay presented a significant reduction standard mean difference (Std. MD) = −2.39 (−3.89, −0.89), *p* = 0.002. The analysis of overall morbidity, specific complications and Clavien-Dindo classification showed no significant variations among the study groups. ERAS protocol in bariatric surgery leads to the reduction of the length of hospital stay while maintaining no or low influence on morbidity.

## Introduction

Obesity is a worldwide issue and its prevalence is growing every year. Bariatric surgery as a method of treatment has become an established and renowned therapy for the management of patients with morbid obesity. The expanding popularity of surgical therapy for morbid obesity has led to an increase in the awareness of the peculiar challenges that bariatric patients pose both to anaesthesiologists and surgeons [1]. Although bariatric surgery has been introduced in the late 1950s, the use of minimally invasive surgery had the most significant impact on improving outcomes. Currently, the laparoscopic approach is the method of choice, with only a small percent of all procedures performed from open access [2]. The most commonly performed surgeries nowadays are laparoscopic sleeve gastrectomy and laparoscopic Roux-en-Y gastric bypass.

Moreover, in the late 1990s, Khelet et al. published a series of papers on enhanced recovery after surgery (ERAS) multimodal programme in colorectal surgery, which has been shown to further reduce complications and shorten the length of stay (LOS) [3]. Subsequently, this idea evolved into a multidisciplinary instrument integrating several perioperative elements which is now recognized as the ERAS protocol with a number of official ERAS Society Guidelines for Perioperative Care in Bariatric Surgery. Several meta-analyses comprising other surgical disciplines documented the benefits of ERAS [4–6].

Most of the items included in the guidelines adopted perioperative elements used widely in other types of surgery [7]. Although there are several studies documenting the feasibility of ERAS in bariatric surgery, the evidence is limited since so far there has been no meta-analysis on this matter. Therefore, our study aimed to systematically evaluate and conduct a meta-analysis of the available evidence on ERAS pathways compared with traditional perioperative care patients undergoing bariatric surgery.

## Methods

### Search Strategy

A search was conducted by two researchers (PM and MW) in April 2016 of Medline, Embase, PubMed, OVID and the Cochrane library, covering a period from January 1966 to May 2016, with the language restricted to English, and using the search terms “bariatric surgery”, “bariatrics”, “metabolic surgery”, “weight loss surgery”, “sleeve gastrectomy”, “gastric bypass”, “gastric banding”, “biliopancreatic diversion”, “duodenal switch”, “omega switch”, “vertical banded gastroplasty”, “sleeve resection” and combinations of these with “fast track”, “enhanced recovery”, “clinical pathway”, “critical pathway”, “multimodal perioperative” and “perioperative protocol”, using the Boolean operators “AND” and “OR”. Reference lists of relevant publications were assessed for additional references. Furthermore, bibliographies from other systematic reviews or meta-analyses on the subject were searched.

A paper was included when the study concerned adult patients who underwent bariatric surgery, the study described an enhanced recovery programme with at least four different perioperative elements according to the guidelines by Thorell et al. [7] or the study reported at least the LOS and the overall complication rate. The papers included had to be either a randomized controlled trial (RCT) or a comparative study with a control group. All criteria mentioned above were required to enrol a study for further evaluation. The exclusion criteria were: the study described a single intervention in perioperative care, the study was a review, guidelines or single group or the study was not in English.

Two researchers (PM and MW) identified and selected citations from the search independently. In the event of uncertainties relating to inclusion, a third reviewer was consulted (MP) until consensus was reached. Data from the included studies were extracted independently by the two researchers. Randomized as well as nonrandomized studies were eligible as long as they met the inclusion criteria. The Jadad scale was used for the quality assessment of the RCTs, which contained randomization (0–2 points), blinding of the studies (0–2 points) and withdrawals (0–1 point). Observational studies were evaluated by the Newcastle–Ottawa Scale (NOS), which consists of three factors: patient selections, comparability of the study groups and the assessment of outcomes. The quality score is presented in Table 1. Missing data were obtained by contacting the authors of the respective studies. The study risk of bias was assessed by visually inspecting funnel plots, using an analytical appraisal based on Egger’s regression test. According to the Egger or Begg methods for publication bias evaluation, two-sided *p* ≤ 0.05 was regarded as significant, in accordance to Egger or Begg methods for publication bias evaluation.

**Table 1:** Study characteristics and quality assessment

Study	Type of surgery	Type of study	No. of patients in study/control group	JADAD/NOS quality score	ERAS elements
Ronellenfitsch et al. [8]	SG, GB, BPD	CS	65/64	6	8
Proczko et al. [9]	SG, GB, rev	CS	146/228	6	6
Pimenta et al. [10]	SG	RCT	10/10	3	4
Petrick et al. [11]	GB	CS	1184/429	5	4
Mannaerts et al. [12]	SG, GB, rev	CS	1313/654	6	15
Lemanu et al. [13]	SG	RCT	40/38	3	13
Geubbels et al. [14]	GB	CS	360/104	6	9
Dogan et al. [15]	GB	CS	75/75	5	12
Campillo-Soto et al. [16]	GB	CS	70/49	6	11
Cooney et al. [17]	GB	CS	12/16	6	9
Barreca et al. [18]	SG, GB	CS	200/88	5	12

*SG* sleeve gastrectomy, *GB* gastric bypass, *rev* revisionary surgery, *BPD* biliopancreatic diversion, *CS* comparative study, *RCT* randomized control trial

### Outcome Measures

The primary outcome measure of this systematic review was the length of hospital stay. Secondary outcome measures were overall morbidity and specific complication rates (bleeding, leakage, cardiopulmonary), mortality and readmissions. Since the Clavien-Dindo (CD) classification was present in five papers, it was used as the primary complication classification [9, 11, 12, 14, 18, 19]. Complications were classified as minor for grade 1 and grade 2 in CD. Complications rated as CD grade 3 and higher were considered major. A study by Dogan et al., in which complications were stratified into minor/major and no CD was used, was also included in the meta-analysis of minor and major complications [15]. We analysed bleeding and suture line leakage, if available, because of its relatively high occurrence in bariatric surgery. The analysis of cardiopulmonary complications was performed to present ERAS influence on nonsurgical complications associated with operation. Furthermore, we also analysed total hospital costs if available.

### Statistical Analysis

The analysis was performed using RevMan 5.3 (freeware from the Cochrane Collaboration). Statistical heterogeneity and inconsistency were measured using Cochran’s *Q* tests and *I* ^2^, respectively. Qualitative outcomes from individual studies were analysed to assess individual and pooled risk ratios (RR) with pertinent 95 % confidence intervals (CI) favouring the ERAS treatment over non-ERAS and by means of the Peto fixed-effects method in the presence of low or moderate statistical inconsistency (*I* ^2^ ≤ 10 %) and by means of a random-effects method (which better accommodates clinical and statistical variations) in the presence of high statistical inconsistency (*I* ^2^ > 10 %). When a study included medians and interquartile ranges, we calculated the mean ± SD using a method proposed by Hozo et al. [20]. Weighted mean differences (WMD) with 95 % CI are presented for quantitative variables using the inverse variance fixed-effects or random-effects method. Statistical significance was observed with two-tailed 0.05 level for hypothesis and with 0.10 for heterogeneity testing, while unadjusted *p* values were reported accordingly. This study was performed according to the Preferred Reporting Items for Systematic reviews (PRISMA) guidelines and MOOSE consensus statement [21].

## Results

The initial reference search yielded 1151 articles. After removing 252 duplicates, 862 articles where evaluated through titles and abstracts. This produced 37 papers suitable for full-text review. As many as 16 articles were excluded due to an incorrect study design, 6 because of an incorrect type of intervention, 2 had a wrong setting, 1 was on non-adult population and 1 was not in English. Finally, we enrolled two RCTs and nine comparative studies with a total of 5230 patients (3475 ERAS and 1755 traditional protocols) of whom 576 underwent bariatric surgery as an open procedure (Table 1) [8–18]. The quality analysis with the Jadad scale for RCTs and NOS scale for comparative studies revealed that both RCTs and comparative studies are of moderate quality, with the majority scoring 6 points for comparative studies and 3 points for RCTs.

**Fig. 1:**
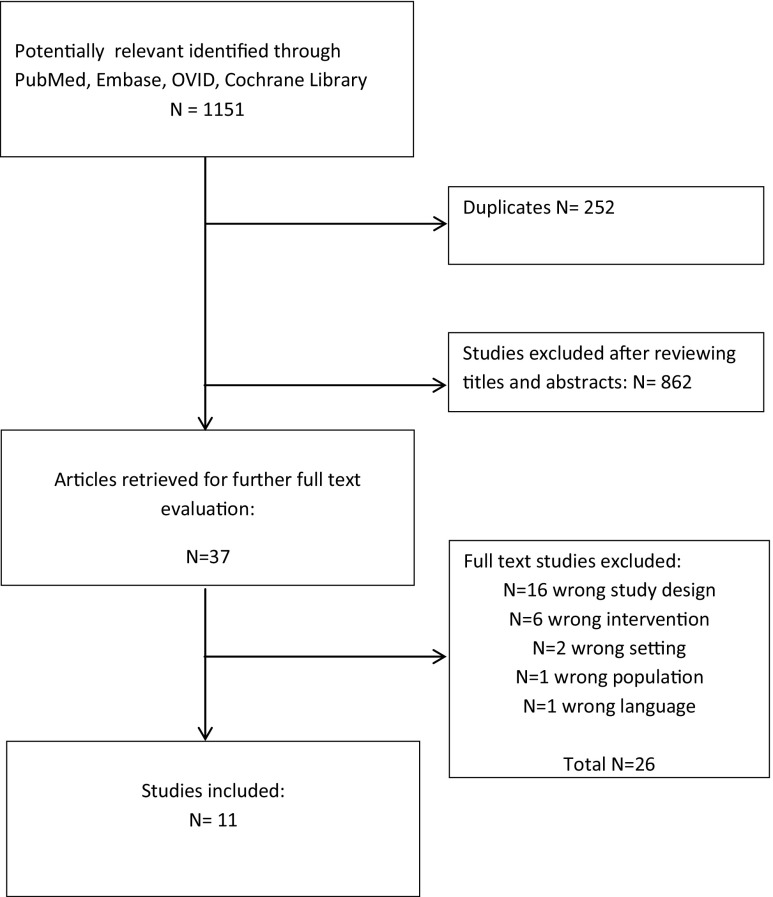
Study selection flow chart

The authors Barreca et al., Mannaerts et al. and Petrick et al. were contacted to acquire additional information. The flowchart of the literature search and study selection is summarized in Fig. 1. ERAS elements used in each study are presented in Table 2.

**Table 2:** ERAS protocol elements

Study	Preoperative counselling	Reduce fasting times	Optimize operating scheduling times	Optimize anaesthesia protocols	Multimodal analgesia	Avoidance of nasogastric tubes and intraabdominal drains	Avoidance of high intraabdominal pressure during leak tests	Early mobilization
Ronellenfitsch et al. [8]	Yes	No	No	Yes	Yes	Yes	No	Yes
Proczko et al. [9]	No	No	No	Yes	Yes	No	No	Yes
Pimenta et al. [10]	No	Yes	No	No	No	No	No	No
Petrick et al. [11]	Yes	No	No	Yes	No	No	No	No
Mannaerts et al. [12]	Yes	Yes	Yes	Yes	Yes	Yes	No	Yes
Lemanu et al. [13]	Yes	Yes	Yes	Yes	Yes	Yes	No	Yes
Geubbels et al. [14]	No	No	Yes	Yes	Yes	Yes	No	Yes
Dogan et al. [15]	Yes	Yes	Yes	Yes	Yes	Yes	No	Yes
Campillo-Soto et al. [16]	Yes	Yes	Yes	Yes	Yes	No	No	Yes
Cooney et al. [17]	Yes	Yes	Yes	Yes	Yes	No	No	Yes
Barreca et al. [18]	Yes	Yes	Yes	Yes	Yes	Yes	No	Yes
Study	Analgesia	Antiemetic	IPP/H2 antagonist	Early enteral feeding	Rigorous blood sugar control	Discharge planning	Follow-up telephone call the day after discharge	Postoperative appointment 2 weeks after discharge
Ronellenfitsch et al. [8]	No	No	No	Yes	No	Yes	No	Yes
Proczko et al. [9]	Yes	Yes	Yes	No	No	No	No	No
Pimenta et al. [10]	Yes	Yes	No	No	No	Yes	No	No
Petrick et al. [11]	No	No	No	No	Yes	No	No	Yes
Mannaerts et al. [12]	Yes	Yes	Yes	Yes	Yes	Yes	Yes	Yes
Lemanu et al. [13]	Yes	Yes	No	Yes	No	Yes	Yes	Yes
Geubbels et al. [14]	Yes	No	No	Yes	No	Yes	No	Yes
Dogan et al. [15]	Yes	Yes	Yes	Yes	No	Yes	No	No
Campillo-Soto et al. [16]	Yes	Yes	Yes	Yes	No	Yes	No	No
Cooney et al. [17]	Yes	No	No	Yes	No	Yes	No	No
Barreca et al. [18]	Yes	Yes	Yes	Yes	No	No	Yes	No

**Fig. 2:**
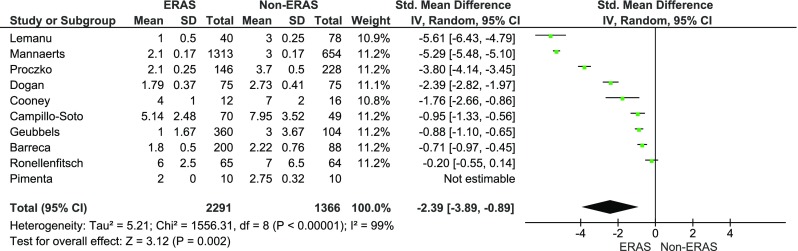
Pooled estimates of the length of hospital stay comparing enhanced recovery after surgery vs. standard care. *CI* confidence interval, *df* degrees of freedom

The mean LOS was reported in all papers and in all of them it included primary LOS (excluding potential readmissions). Petrick et al. did not provide information on SD for LOS; thus, this paper was excluded from the meta-analysis of LOS. There was a significant reduction in LOS in all papers but one, by Ronellenfitsch et al. [8]. The mean LOS for the ERAS group was 2.8 days, while for the control group it was 4.6 days. The analysis (Fig. 2) showed significant differences between the studied groups: standard mean difference (Std. MD) = −2.4, 95 % CI −3.9 to −0.9, *p* for effect = 0.002, *p* for heterogeneity <0.00001, *I* ^2^ = 99 %.

**Fig. 3:**
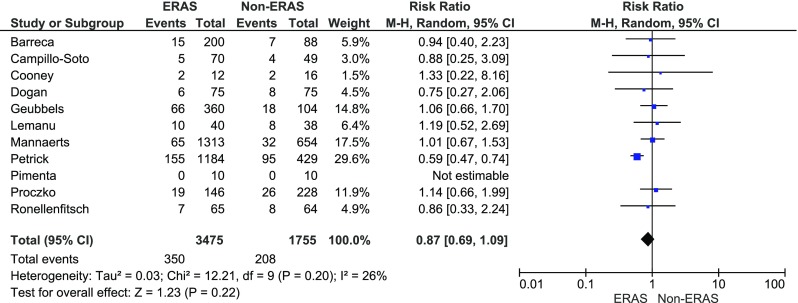
Pooled estimates of overall morbidity comparing enhanced recovery after surgery vs. standard care. *CI* confidence interval, *df* degrees of freedom, *RR* risk ratio

The overall morbidity was reported in all studies. In ten studies, there were no statistical differences in the complication rate. One study by Petrick et al. favoured the ERAS group [11]. Pimenta et al. reported no complications in both groups [10]. The meta-analysis of the included studies showed that the overall morbidity between the studied groups did not vary significantly: 350/3475 (10.1 %) in ERAS group vs. 208/1755 (11.9 %) in the control group, RR = 0.9, 95 % CI 0.7–1.1, *p* for effect = 0.2, *p* for heterogeneity = 0.2, *I* ^2^ = 26 % (Fig. 3).

**Fig. 4:**
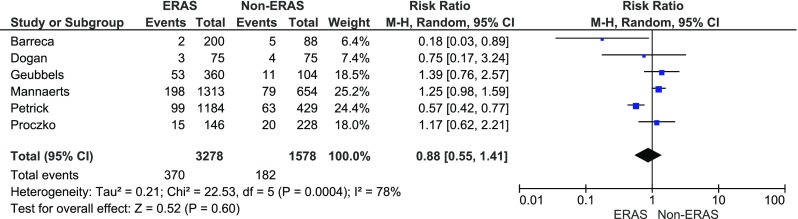
Pooled estimates of minor complications comparing enhanced recovery after surgery vs. standard care. *CI* confidence interval, *df* degrees of freedom, *RR* risk ratio

**Fig. 5:**
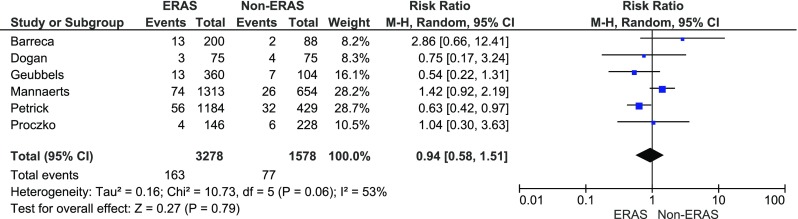
Pooled estimates of major complications comparing enhanced recovery after surgery vs. standard care. *CI* confidence interval, *df* degrees of freedom, *RR* risk ratio

In five papers, complications were presented using the CD classification. Grades 1 and 2 were stratified as CD minor, while grade 3 and above as major. Dogan et al. reported complications as major and minor which were also included in the meta-analysis of minor and major complications [15]. There were no significant variations among the studied groups in minor complications: 370/3278 (11.3 %) in ERAS group vs. 182/1578, (11.5 %) in control group, RR = 0.9, 95 % CI 0.6–1.4, *p* for effect = 0.6, *p* for heterogeneity = 0.0004, *I* ^2^ = 78 % (Fig. 4). The analysis of major complications (Fig. 5) also showed no differences between the groups: 163/3278 (5 %) in ERAS group vs. 77/1578 (4.9 %) in control group, RR = 0.9, 95 % CI 0.6–1.5, *p* for effect = 0.8, *p* for heterogeneity = 0.1, *I* ^2^ = 53 %.

**Fig. 6:**
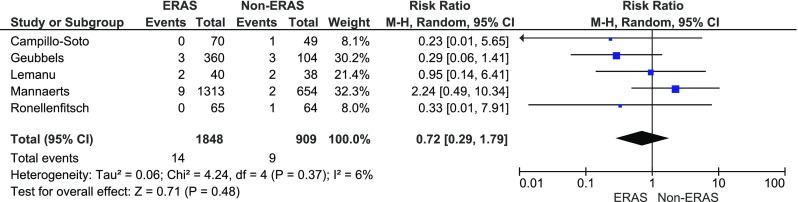
Pooled estimates of suture line leakage events comparing enhanced recovery after surgery vs. standard care. *CI* confidence interval, *df* degrees of freedom, *RR* risk ratio

The suture line leakage rate was reported in five papers. The analysis (Fig. 6) showed no significant variations among the studied groups: 14/1848 (0.8 %) in ERAS group vs. 9/909 (1 %) in control group, RR = 0.7, 95 % CI 0.3–1.8, *p* for effect = 0.7, *p* for heterogeneity = 0.4, *I* ^2^ = 6 %.

**Fig. 7:**
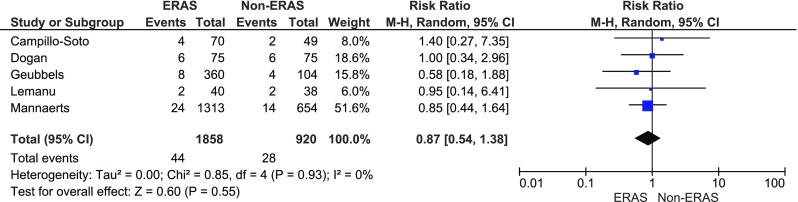
Pooled estimates of bleeding events comparing enhanced recovery after surgery vs. standard care. *CI* confidence interval, *df* degrees of freedom, *RR* risk ratio

Bleeding was reported in five papers. The analysis (Fig. 7) showed no significant variations among the studied groups: 44/1858 (2.4 %) in ERAS group vs. 28/920 (3 %) in control group, RR = 0.9, 95 % CI 0.5–1.4, *p* for effect = 0.6, *p* for heterogeneity = 0.9, *I* ^2^ = 0 %.

**Fig. 8:**
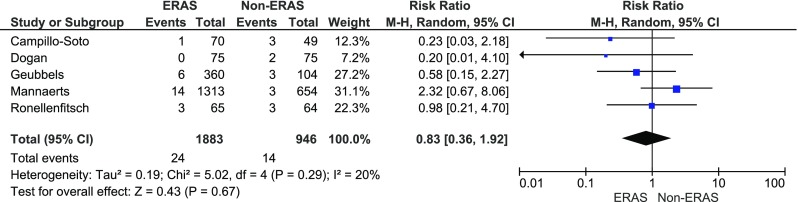
Pooled estimates of cardiopulmonary complications comparing enhanced recovery after surgery vs. standard care. *CI* confidence interval, *df* degrees of freedom, *RR* risk ratio

Cardiopulmonary complications were reported in five papers. The analysis (Fig. 8) showed no significant variations among the studied groups: 24/1883 (1.3 %) in ERAS group vs. 14/946 (1.5 %) in control group, RR = 0.83, 95 % CI 0.4–1.9, *p* for effect = 0.7, *p* for heterogeneity = 0.3, *I* ^2^ = 20 %.

Data on mortality was presented in eight articles. Petrick et al. reported general mortality of 0.7 % with no division into ERAS and non-ERAS. The analysis of data from eight articles presented no differences in mortality between the ERAS group (1/2006; 0.05 %) vs. control group (3/1163; 0.26 %) (RR = 0.4, 95 % CI 0.1–2.2, *p* for effect = 0.3, *p* for heterogeneity = 0.8, *I* ^2^ = 0 %).

**Fig. 9:**
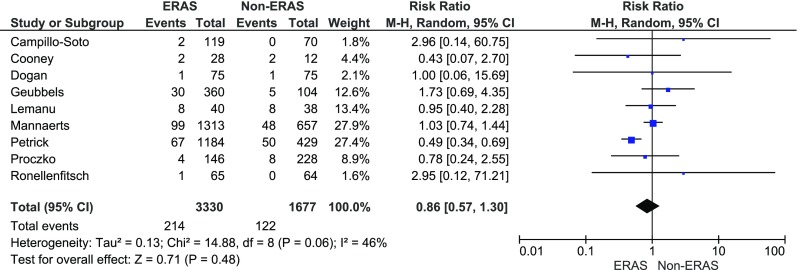
Pooled estimates of hospital readmission comparing enhanced recovery after surgery vs. standard care. *CI* confidence interval, *df* degrees of freedom, *RR* risk ratio

Readmission rate was provided in nine studies. The analysis (Fig. 9) presented a tendency of lower rate in the ERAS group (214/3300; 6.5 %) than in the control group (122/1677; 7.3 %) (RR = 0.9, 95 % CI 0.6–1.30, *p* for effect = 0.5, *p* for heterogeneity = 0.06, *I* ^2^ = 46 %).

**Fig. 10:**
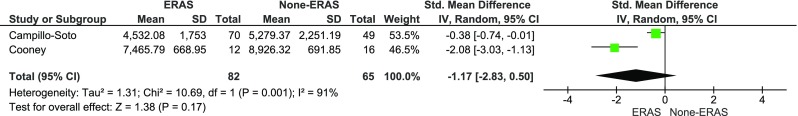
Pooled estimates of treatment costs comparing enhanced recovery after surgery vs. standard care. *CI* confidence interval, *df* degrees of freedom

Cost difference was reported by Campillo-Soto et al. and Cooney et al. [16, 17]. The analysis (Fig. 10) showed no significant cost reduction between the studied groups: Std. MD = −1.2, 95 % CI −2.8–0.5), *p* for effect = 0.2, *p* for heterogeneity = 0.001, *I* ^2^ = 91 %.

## Discussion

This systematic review, based on 1151 articles, establishes that ERAS protocol in bariatric surgery is a safe and feasible method of perioperative care. After thorough research, the final analysis involved 11 studies of which two were RCT and nine were clinical control studies. The analysis showed a general reduction of LOS with no significant influence on the overall morbidity or specific complication rates and a tendency to reduce readmission rate. Furthermore, the introduction of ERAS protocol may be associated with general cost reduction. Using a standardized perioperative protocol (not necessarily called ERAS), leading to ultra-short LOS, even without an overnight stay, has been previously shown as feasible in case series studies [22, 23]. These authors emphasize the importance of a well-defined standardized perioperative protocol, the use of laparoscopic surgery, a properly educated medical team and, most importantly, appropriate patient education and compliance. Moreover, in their opinion, careful patient selection and precise guidelines may contribute to maximize patient safety and outcomes. Although these papers had been published before ERAS guidelines were formulated, they include many aspects which are covered in 2016 ERAS guidelines for bariatric surgery.

The efficacy of ERAS protocol in surgery was confirmed in previous systematic reviews regarding colorectal, pancreatic, gastric or liver surgery [4–6, 24]. These studies showed the reduction of LOS and a decrease in complication rate. The number of studies regarding the application of ERAS protocol in bariatric surgery is limited. In our literature research, we came upon two systematic reviews by Elliott et al., published in 2013, and by Lemanu et al., published in 2012 [25, 26]. These papers did not include any comparative studies or meta-analysis; thus, the quality of evidence was limited. Our systematic review is based on comparative studies with subsequent meta-analysis conducted on the data provided. Moreover, nine studies used in this analysis were published after the latest review.

While ERAS protocol led to the reduction of LOS and complication rate in different surgical disciplines, we found that in bariatric surgery only LOS was affected [4–6]. This effect was observed regardless of the surgery type. In the case of ERAS pathways in bariatric surgery, the reduction of the length of stay may be due to clearly defined discharge criteria, which may decrease the possibility of patients staying longer than required. We showed that morbidity in ERAS after bariatric surgery was similar in both the ERAS group and the control group. Additionally, no influence of the type of surgery was noted. Therefore, it is reasonable to assume that its introduction is safe, regardless of the type of operation. The lack of differences may be caused by the low complication rate in bariatric surgery in general, which is lower than in other fields of surgery. Furthermore, the reduction of LOS while having similar complication rates allows the assumption that ERAS protocol improves functional recovery. It is believed that modern perioperative care leads to the reduction of postoperative stress response, thus allowing faster convalescence [27]. According to the ERAS Congress held in Cannes in 2013, full functional recovery after surgery is the main goal of perioperative care [28]. The limitation to this statement is the inconsistency of how complications were reported in the analysed studies. Apart from the studies which described them in Clavien-Dindo classifications, the severity of bleeding or leakage was not determined, making it impossible to present a unified, reliable analysis of complications. Therefore, the quality of evidence provided in the included articles is rather low. Based on two studies, by Campillo-Soto et al. and Cooney et al., we showed that ERAS may be cost-effective [16, 17]. The decreased costs in the case of ERAS protocol mostly result from the reduced LOS and the tendency of lower readmission rate. Similar conclusions were drawn by Joliat et al. and Lemanu et al. in colorectal surgery and pancreatic surgery [29, 30].

**Fig. 11:**
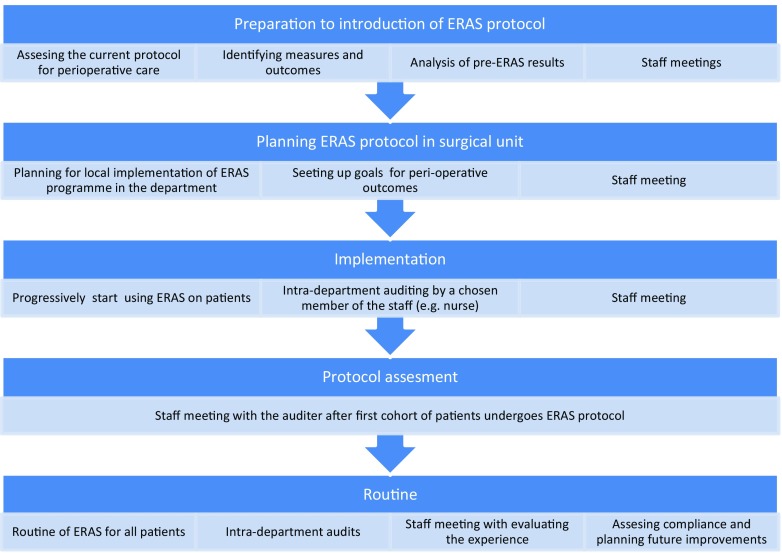
Protocol implementation pathway based on ERAS Society programme

Perhaps the greatest difficulty regarding ERAS protocol is its implementation. Since it stands in opposition to a number of surgical dogmas, it may be considered more of a revolution than an evolution in perioperative care. Therefore, ERAS Society has introduced a training programme designed for perioperative care teams to implement various ERAS protocols for different surgical fields, including bariatrics. It consists of series of workshops and seminars over the period of 8–10 months that are conducted by an ERAS coach and a medical expertise from ERAS Society (Fig. 11). Moreover, a specially designed interactive auditing database is used to fully assess the overall adherence to the ERAS protocol and to each particular item. Continuous auditing is the key to achieving high level of compliance and helps in early identification of any potential deviation from the protocol. All of these result in an improvement of the outcomes [31, 32].

We included studies presenting data both regarding laparoscopic as well as open approach to bariatric surgery. The rate of open cases (11 %) is different from the most recent reports from the USA (more than 95 % procedures are performed laparoscopically). It is well established that the open approach is associated with a higher morbidity rate and longer LOS than laparoscopy; thus, it may raise concerns regarding bias. However, the aim of our study was to determine the influence of ERAS protocol on bariatric surgery in general, due to the fact that open approach is still performed in some countries [11, 33]. Moreover, initially the idea of ERAS has been developed for open surgery; therefore, we think that including open cases seems reasonable.

The quality of the papers included is limited. Only two of the analysed studies were RCT, whereas the remaining were clinical control studies. Due to the nature of ERAS protocol itself, it is difficult to perform a full RCT. Both patients as well as physicians are required to act according to certain requirements of this pathway, thus resulting in the lack of blinding of the RCT. This was mentioned in the study by Lemanu et al. [13]. Greco et al. [6] concluded that the evidence proving feasibility and efficacy of ERAS protocol in colorectal surgery is so strong that performing randomized trial on patients may be considered as providing insufficient health care to the patients in the control arm. None of the analysed studies used all ERAS elements presented in the ERAS guidelines [7]. It has to be emphasized that the variability in both the number and the type of ERAS items implemented did not permit a reliable subgroup analysis to identify which items might be more effective. We did not find any link between the number of ERAS elements and the reduction of LOS. For instance, Mannaerts et al. with 15 ERAS elements had similar LOS reduction (30 %) as Petrick et al. with only four elements (30.1 %). Unfortunately, compliance with the protocol was only reported by Campillo-Soto et al., Lemanu et al. and Petrick [11, 13, 16]. This is important, since many papers link adherence to the protocol with postoperative outcome [34, 35]. Although there are items that theoretically have greater influence on outcomes (laparoscopic surgery, fluid management, early feeding and ambulation), the success of ERAS programmes resembles improvements described by Sir David Brailsford, performance director of GB cycling team, as aggregation of marginal gains theory [36]. He described the principle of multiple improvements throughout any given process, collectively achieving a far superior output. Due to the lack of data in other studies, it is impossible to determine the compliance rate in this review. The lack of ERAS compliance reporting, a different number of protocol elements, heterogeneity of the studies and no unified stratification of morbidity classification prevent us from making strong conclusions about ERAS in bariatric surgery. The first ERAS Society Guidelines for Perioperative Care in Bariatric Surgery were published in 2016, meaning that all included studies did not use them in the design of a standardized protocol. Therefore, future studies on adherence to these new recommendations and the influence of protocol compliance on outcomes may allow further investigation on ERAS in bariatrics.

## Conclusion

This is the first systematic review with a meta-analysis on ERAS in bariatric surgery indicating a reduction in the length of hospital stay with no influence on morbidity. There is also a tendency of readmission reduction as well as cost reduction. Most of the papers analysed were comparative studies with a high risk of bias. Although we do not believe more RCTs are necessary to show benefits of ERAS, further research on the compliance with the protocol is required to fully assess the feasibility of modern peri-operative care protocols in bariatric surgery.
